# Caring animals and the ways we wrong them

**DOI:** 10.1007/s10539-023-09913-1

**Published:** 2023-06-27

**Authors:** Judith Benz-Schwarzburg, Birte Wrage

**Affiliations:** Messerli Research Institute, University of Veterinary Medicine Vienna, Vienna, Austria

**Keywords:** Animal care behavior, Empathy, Moral care, Care ethics, Animal welfare, Objective value

## Abstract

Many nonhuman animals have the emotional capacities to form caring relationships that matter to them, and for their immediate welfare. Drawing from care ethics, we argue that these relationships also matter as objectively valuable states of affairs. They are part of what is good in this world. However, the value of care is precarious in human-animal interactions. Be it in farming, research, wildlife ‘management’, zoos, or pet-keeping, the prevention, disruption, manipulation, and instrumentalization of care in animals by humans is ubiquitous. We criticize a narrow conception of welfare that, in practice, tends to overlook non-experiential forms of harm that occur when we interfere with caring animals. Additionally, we point out wrongs against caring animals that are not just unaccounted for but denied by even an expansive welfare perspective: The instrumentalization of care and caring animals in systems of use can occur as a harmless wrong that an approach purely focused on welfare may, in fact, condone. We should therefore adopt an ethical perspective that goes beyond welfare in our dealings with caring animals.

## Introduction

Many nonhuman animals (henceforth ‘animals’) possess complex socio-emotional capacities and thus can have relationships that *matter to them* in their particularity, characterized by empathic care. Care in these relationships is not provided as an automatic, non-voluntary stimulus response, but is emotionally motivated and flexible, and thus can be credited with some non-reflective intentionality.^1^ It is this capacity to care, and these relationships of care that animals are emotionally invested in, that we are interested in here. These are most paradigmatically parent–child relationships (Decety et al. 2016), in which empathic care is understood as a basic strategy to increase the chances that one’s children survive (Pianka 1970). Empathy as an emotional sensitivity to others’ needs is a powerful proximate motivator for care, and therefore thought to be evolutionary old and relatively widespread, at least in birds and mammals (de Waal 2008, 279). Moreover, empathic care occurs beyond the parent–child relationship among relatives, friends, and other community members. Our considerations therefore concern a wide range of species, and many social contexts.

In humans, caring relationships are valued as fundamental to our existence as the kind of social and interdependent beings we are. Loneliness takes a significant toll on our health including mental health (Park et al. 2020), and social deprivation in sensitive developmental periods leaves humans emotionally scarred for life (e.g. Nelson et al. 2019). Ostracizing a member of the community may be one of the worst punishments, and prolonged social confinement is regarded as a form of torture (UNODC 2015, 14). In turn, social relationships and social support increase individual resilience in the face of trauma (e.g. Ponce-Garcia et al. 2015), and social relationships are the strongest protective factor for depression (Choi et al. 2020). The Harvard Study of Adult Development, a longitudinal study of adult life that has been running for 80 years, found that good relationships are a key factor in living longer and happier lives (see Mineo 2017 for an overview).

In addition to this fundamental role for our wellbeing, caring relationships have been emphasized in feminist moral theory, especially care ethics, as basic to our *moral* lives (e.g. Noddings 1984/2013; Baier 1987/2002; Held 1993; 2006; Tronto 1993). In this tradition, caring about others emotionally is viewed as the wellspring of moral concern, and caring for others is understood as a fundamental moral practice that enables and sustains morality, and is itself the correct moral response to our fundamental interdependence. We need care to become the kinds of moral beings we are, and care is a fundamental expression of the kinds of moral beings we are. Without care, we are fundamentally altered, and if we did not need care, we would be fundamentally different kinds of moral beings, because we would not have this specific vulnerability that appeals to morality.

Although humans are not unique in their fundamental dependence on care, we note a substantial lack of human concern for caring relationships in animals. This is evident across all major contexts of human-animal interaction. Be it in farming, animal experimentation, wildlife ‘management’, zoos, or pet-keeping, the prevention, disruption, manipulation and instrumentalization of care in animals by humans is ubiquitous. Stalls and tethers on farms, social isolation and maternal deprivation in labs, or hand-rearing in zoos are just a few examples for procedures that deeply impact caring relationships in animals.

In this paper we argue that such treatment not only negatively affects animals’ wellbeing, leading to *harm-based wrongs*, but also disregards the objective value of care, leading to *non-harm-based wrongs*. Harm-based wrongs comprise damage to *immediate welfare* but also the *deprivation of (future) goods arising from caring relationships*, like trust and friendship, to the extent that such goods bare *instrumental value* for wellbeing. Harm-based wrongs are *experiential* in nature and the ultimate entities affected by them are the subjects engaged in caring behavior, which is why we speak of the *subjective value of care* being violated. Harm-based wrongs can be addressed from a broad welfare perspective, the view that experiential wellbeing, however understood, is the only moral value. Non-harm-based wrongs, in contrast, are not covered by a welfare perspective. They are *non-experiential* in nature and *damage other values than wellbeing*, in our case the value of care itself, some value assigned to the caring agent, or the value of caring relationships. Because this wrong cannot be captured in terms of subjective welfare we speak of the *objective value of care* being disregarded. We argue that a recognition of the objective value of care in animals is stringent, based on the way in which the value of care can be argued as subjective and objective, but not anthropogenic.

Acknowledging the value of care in animals has serious ethical implications from the perspective of animal welfare ethics, because humans regularly interfere with caring animals in harmful ways. However, if we approach interferences with caring animals from a mere welfare perspective, non-harm-based wrongs are likely left unaddressed. In fact, we find that a focus on welfare alone may even lead to an endorsement of the instrumentalization of care and caregivers in fundamentally uncaring systems, like the dairy and meat industry. To show these limitations of welfarism as such we stress non-harm-based wrongs arising from the interference with caring animals and their relationships. We argue the objective value of care in animals by help of care ethical theory: Caring animals contribute to a morally better world in which vulnerability is met by care, not by ignorance. This contribution has more than subjective value.

Care in animals is a multiply neglected but pressing issue. On the one hand, animal ethics has long focused on sentience as the central capacity relevant for moral status, leading to a focus on harm-based wrongs in animal ethics. But the political turn in animal ethics has recently shifted the focus on animals’ agency, including their socio-cognitive and emotional capacities, pointing to the just inclusion and political participation we owe other animals (e.g. Donaldson and Kymlicka 2011; Meijer 2019; Benz-Schwarzburg 2020). If animals’ caring relationships possess subjective as well as objective value, they may be one highly relevant piece of the puzzle of a just zoodemocracy and contemporary animal ethics, as caring animals are not only hindered in their relationships, but also, like humans, instrumentalized and exploited as caregivers.

On the other hand, care ethics with its focus on care as a crucial complement to justice considerations is still a marginalized philosophy in general.^2^ Accordingly, it is no surprise that care ethics is also marginalized in the animal ethics debate in specific. In addition, so far, animal ethicists in the care ethical tradition mainly focus on caring relationships between *humans* and animals, with humans as caregivers (e.g. Donovan and Adams 1996, 2007; Gruen 2015). This means that care ethics has recognized animals as potential *recipients of human care*, but has not fully explored their status as potential *caregivers* in their own right (Wrage 2022). However, there does not appear to be an obvious reason why the value of care should be tied to species membership. Ultimately, as we will show, the subjective value of care is widespread in nature, and the objective value of care is universal, not limited to humans. This puts animals’ *own* relationships on the map of animal ethics.

To substantiate and motivate our assertion that animals have morally relevant relationships of care, we briefly review the available empirical literature on empathic care in animals (Sec. "Empirical data on empathic care in animals"). We then elaborate on the value of care, and distinguish between its subjective and objective value (Sec. "The value of care"). In Sect. "Harm-based and non-harm-based wrongs in our treatment of caring animals", we consider wrongs resulting from the neglect of either dimension of value of care in animals, looking at human interference with animals’ *exercise of care behavior* (Sec. “Human interference with animals’ exercise of care behavior”), with (the development of) the animals’ *capacity to care* (Sec.” Human interference with animals’ capacity to care”), and cases of human *instrumentalization of care* in animals (Sec. “Emphasizing non-harm-based wrongs against caring animals”). We contrast subjective welfare considerations with the additional moral concerns that a care ethical perspective introduces by emphasizing non-harm-based wrongs that occur independent of welfare harms, sometimes due to interventions *meant to improve* welfare. We thereby identify moral wrongs against caring animals that a purely welfare-oriented approach, even a comprehensive one, fails to recognize as such.

On a related note, we have to concede that some of the research we cite in this paper to substantiate the value of animals’ relationships itself undermines this value. We refer to this research with regret and hope that this paper makes salient why such disregard for animals’ relationships is ethically problematic.

## Empirical data on empathic care in animals

To what extent do animals have caring relationships that could matter morally? As mentioned in the introduction, we are interested here in caring relationships that animals are emotionally invested in, that they care *about*, not in care behavior that is an involuntary fixed behavior pattern. Thus, empathy as an emotional motivation for care is a crucial capacity for an animal to be capable of engaging in such relationships. The capacity for empathy, understood as a sensitivity to other’s needs that *motivates care*, is taken to be phylogenetically old and widespread in animals (e.g. de Waal 2008, 279). Parental care in particular is a prime context in which a sensitivity to others’ needs, accompanied by a motivation to care for them, would evolve, as it increases offspring survival (Decety et al. 2016). Indeed, emotional contagion as a basic form of or precursor to empathy has been found, for example, in chickens (Edgar et al. 2011), chimpanzees (Parr 2001), dogs (Quervel-Chaumette et al. 2016; van Bourg et al. 2020), geese (Wascher et al. 2008), mice (Langford et al. 2006; Jeon et al. 2010), pigs (Reimert et al. 2015; Goumon and Špinka 2016), prairie voles (Burkett et al. 2016), and rats (Knapska et al. 2006). More complex forms of empathy that involve a degree of perspective-taking have been claimed for cows (Ede et al. 2020, 7), cetaceans (see Pérez-Manrique and Gomila 2018 for a review), some nonhuman primates (ibid.), and elephants (Bates et al. 2008). This means that we find the capacity to be emotionally motivated to care, and thus to care with some intentionality, beyond a fixed stimulus–response, in a range of animals.

In addition to empathic parental care there are two forms of empathic care of which we have substantial empirical data in animals. First, empathic helping has been observed in bonobos (Melis 2018), chimpanzees (Yamamoto et al. 2012), dogs (Sanford et al. 2018; van Bourg et al. 2020), dolphins (Park et al. 2013), elephants (Bates et al. 2008), humpback whales (Pitman et al. 2017), mice (Ueno et al. 2019), and rats (Ben-Ami Bartal et al. 2011). Second, consolation, an empathically motivated increase in affiliation in response to another’s distress (Burkett et al. 2016, based on de Waal and van Roosmalen 1979), has been observed in dogs (Quervel-Chaumette et al. 2016), dolphins (Yamamoto et al. 2015), corvids (Seed et al. 2007; Fraser and Bugnyar 2010), elephants (Plotnik and de Waal 2014), primates (de Waal and van Roosmalen 1979; Palagi et al. 2004; Cordoni et al. 2006; McFarland and Majolo 2012), and voles (Burkett et al. 2016).

Furthermore, there is some observational evidence of even more exceptional care behavior in animals, such as rescue behavior (e.g. in boars: Masilkova et al. 2021; in elephants: Poole and Moss 2008, 80; Bates et al. 2008, 215), spontaneous foster-parenting of unrelated infants (e.g. Hobaiter et al. 2014) or adoption of heterospecific infants (Izar et al. 2006; Carzon et al. 2019). We also find special care towards injured (e.g. in wild barbary macaques: Campbell 2019; in elephants: Bates et al. 2008, 217f), disabled (in elephants: Bates et al. 2008; in chimpanzees: Bekoff and Pierce 2009, 97), dying or dead conspecifics (in elephants: Douglas-Hamilton et al. 2006) and heterospecifics (bonobo assisting an injured bird: de Waal 2006, 2). In chimpanzees, the tendency to empathize with others has even been found to vary between, and to be stable in individuals long-term, which suggests “empathic personalities” (Webb et al. 2017).

Taken together, the data strongly indicate that animals have caring relationships of the relevant sort, i.e. that matter to them, as the necessary empathic capacity is relatively widespread in nature, and several salient instances of the practice of care, starting with empathic parental care, have been well documented.

## The value of care

Care ethics, an ethical theory that has explored the value of care in detail, views care as a moral value or a “cluster of values” (Held 2006, 4; see also Tronto 1993, 9; Noddings 1984/2013, 84). This view is the basis for the ethical claim that relationships of care and caring beings as such merit protection. In light of the empirical data on care in animals, we want to ask what kind of moral consideration we owe caring animals, what value do their relationships have? To answer this, we distinguish between the ‘subjective’ and the ‘objective’ value of care. Based on this distinction we then consider how current practices of human-animal interaction are wronging caring animals as such. As defined in our introduction we understand the subjective value of care as emerging from the subjective experience of an individual (i.e. the individual in relation experiences the relationship as good, they care about it, it matters to them), and the objective value of care as an external ethical perspective from which we as a moral society value care. What follows from our distinction are two broad clusters of wrongs: ‘harm-based wrongs’ and ‘non-harm-based wrongs’. Harm-based wrongs are experienced as such by the caring or cared-for animals and grounded on the *subjective value* of care for those involved; non-harm-based wrongs, in turn, are grounded on the *objective value* of care and caring relationships, and do not necessarily involve experiential suffering. We argue that especially the objective value of care in animals, and thus non-harm-based wrongs to them, have been neglected so far, as ethical debates on animals tend to focus on experiential welfare.

### The subjective value of care

Care is a moral value, firstly, to the extent that caring relationships have *subjective value, i.e.* “participants take the relationship to be valuable to them,” they *care about it* and even experience it as life-enhancing beyond its mere utility (Collins 2015, 41ff). The fact that care, first of all, *matters* to those involved is an implication of how the moral emotions involved work: empathy, for example, motivates care *for* others (i.e. care as a behavior), because it entails care *about* others (i.e. an emotional motivation). By definition, caring relationships thus matter subjectively to those involved.

But care not only matters on the level of personal motivation, it also *feels good* to give and receive care. Thereby it is, on the one side, *the act of caring itself* which is experienced as rewarding. Scientists speak of the “warm glow” experience (Andreoni 1990): it feels good to care for others and it feels good to be cared for, thus care enhances immediate welfare. On the other side, it is also *the valuable states of affairs created by care* that enhance wellbeing. Care provides unique access to other (subjective) values such as the experience of trust or friendship (Collins 2015, 45), belonging or dependence, and can generate meaning in life beyond its hedonic features. For instance, parents find caring for their children to be more meaningful than their paid work, although they also find it more exhausting (Wang and Pew Research Center 2013). The subjective value of care is attached to relationships in their particularity; for example, a loving parent does not just value having *a* child, but their *particular* child. Ultimately, parental care is likely the blueprint for all our caring: care directed at people other than our children still activates regions of the brain that are usually involved in parental care (Inagaki and Ross 2018).

Taken together, care is subjectively valuable not only for the recipient but also for the caregiver in and outside of parent–child relationships. This has ethical implications. The subjective value of care implies that the thwarting of care likely leads to subjective harms. These harm-based wrongs either consist of immediate suffering (e.g. stress in the context of forced weaning, as is the norm in farming contexts), or of reduced wellbeing due to the deprivation of positive experiences (e.g. lack of deep social bonds due to frequent re-grouping). A broad theory of welfarism will be able to account for both these wrongs, as they undermine wellbeing. Care, from this perspective then, is of *instrumental value* for the wellbeing of a social animal.

We think it is important to see that welfare theories can, and indeed should, assign a crucial role to care in animals due to its instrumental role for wellbeing. Care can, in fact, be viewed as a stringent extension of the non-maleficence principle–it is impossible to avoid harm to beings that depend on care, if there is at the same time no duty to care. Care as the responding to dependency and meeting of others’ needs, in turn, implies non-maleficence and to support flourishing in the sense of wellbeing.

However, care thus understood is a fundamental pillar of ethics in general, not just of welfare ethics. De Grazia (2005) claims that a moral system not adhering to the principle of non-maleficence is a broken one: it would “hardly be recognizable as a moral system”, and anyone “who is neither a nihilist nor a psychopath” usually accepts this principle. Similarly, we think a system of thought or a society of vulnerable beings in which care is not embraced as a value would not only be unjust but inherently immoral.

This connection between care, non-maleficence, and broader ethical perspectives should motivate us to look beyond welfare when determining the value of care in animals. After all, care might not only be of instrumental value for wellbeing but also of inherent value in and of itself. This is important because we also need to define how much weight animals’ care-related interests have. Welfare-based policy usually doesn’t arrive at a strong protection of animals’ interests, but stays at the level of utilitarian harm-benefit analysis. Our following argumentation is an attempt to assign additional weight to care as an absolute value that renders it questionable whether it can be trumped by human interests all too easily. We think indeed, that care can be considered valuable from an external perspective, objectively, which points to the possibility of other kinds of moral wrongs than welfare harm.

### The objective value of care

In what follows we map the objective value of care by focusing on two crucial thoughts: First, we refer to the link between care and vulnerability to argue a meta-ethical conceptualization of care as prima facie duty or objective good. Second, we highlight the special role care ethicists assign to early social relations in ontogeny (Govrin 2014): Social animals depend on *receiving* care for their own *development* of caring capacities, which enable and sustain morality, which is why care ethics views care as fundamental to our moral development. Potentially caring beings need to receive care to, in turn, continue a caring world. We think this grounds the characterization of care as objectively valuable and worthy of protection.

#### Care as prima facie duty and objective good

Care has *objective value*, i.e. it is valuable in and for itself from a broader or more fundamental perspective than that of the individual subject experiencing it (e.g. Gheaus 2009). Applebaum (1998, 420) argues that care is a “prima facie duty”, and that we intuitively acknowledge the goodness of prima facie duties. On this account, “[n]ormativity is conceptually built into our understanding of what it means to care,” and care even retains its inherent goodness regardless of whether it is the best thing to do in a given situation (ibid.). A caring act might be misguided or embedded in an unhealthy relationship (Collins 2015, 40), still, care itself, and the fact that one was moved to care, is good.

The idea of prima facie duties stems from moral philosopher William David Ross who claims that already “at an earlier stage of moral development” it becomes clear to us that “if there are things that are bad in themselves we ought, *prima facie*, not to bring them upon others; and on this fact rests the duty of non-maleficence” (Ross 1930/2002, 26). Besides non-maleficence, other core concepts like fidelity, gratitude, beneficence or justice are on Ross’ list of prima facie duties. A prima facie duty is sensitive to the varying demands of situation and context but obligatory and binding, other things being equal (Potter 2011; Ross 1930/2002). Explaining the objective character of prima facie duties, Ross (1930/2002, 20, our emphasis) states: “What I am speaking of is *an objective fact involved in the nature of the situation*, or more strictly in an element of its nature”. What Ross describes here as an inherent *objective fact involved in the nature of a situation* corresponds to Applebaum’s characterization of the *inherent goodness* of care, or the idea that we *conceptually build normativity into our understanding of care* (Applebaum 1998): we think care is generally the right thing to do, and that it is generally a good thing to care. Care could thus be one among other crucial prima facie duties.

But care could also play a more fundamental role. Philosophers have argued that our world is not only better for containing beings who care (Rowlands 2012, 253f; Applebaum 1998, 419f), but a world without care would be fundamentally at odds with our embodied, vulnerable existence (Houston and Diller 1987, 36, cited after Applebaum 1998, 416). In feminist, care-ethical, virtue-ethical and phenomenological debates scholars as different as Martha Nussbaum (2004; 2007), Judith Butler (2004; 2009), Erinn Gilson (2014), or Alasdair MacIntyre (1999) all point to the fact that humans are fundamentally dependent on others. As embodied and profoundly social beings they are reliant on care, and their vulnerability is understood as a key aspect of the conditio humana. However, some animals plausibly share this kind of vulnerability with humans, in light of their sociality and emotional complexity. Monsó et al. (2018, 294–296) argue, with reference to Martha Nussbaum’s capabilities approach, that moral emotions such as empathy are essential to the flourishing of social and possibly moral animals *as the sort of thing they are* (Nussbaum 2004, 306). Their attachments to others and relationships of care and love are fundamental to *what it means to lead a good life for them* (see, e.g., Nussbaum 2007, 345).

While flourishing thus understood is clearly connected to experiential wellbeing, it is not reducible to it. What we find here as ultimate grounding of value is the ontological characterization of social animals *as caring beings* rather than a welfare idea.^3^ Nussbaum uses the term “flourishing” to locate ethical significance in the existence of complex social forms of life, not just in their wellbeing (Monsó 2018, 294). The thwarting of care potentially leads to a worse life for beings who are caring individuals and depend on care, and again, “worse” is to be understood in more than welfare terms, a life in which they cannot flourish *as the kinds of social beings they are*. Instead of being just one among different prima facie duties, care might in fact be constituent to other prima facie duties like justice. Indeed, Nussbaum embeds her idea of flourishing in a framework of justice (Nussbaum 2004; 2007).

While the subjective value of care is connected to the possibility of subjective harm, i.e. harm experienced as such by the individual, the described objective value of care points to the risk of non-harm-based wrongs when care is disrespected. These wrongs can be identified as objectively morally bad, no matter if any affected individual knows it or experiences them as harmful. Imagine, for example, we could take away the ability to care from an otherwise caring individual, and imagine we did this non-invasively, without causing any suffering. Furthermore, the individual might not even realize that their ability to care is gone. Still, our characterization of care as a prima facie duty, something we intuitively grasp to be right and good, speaks to a moral wrong being done here. An objective good has been destroyed, because a formerly caring individual is precluded from engaging in caring relationships.

Non-harm-based wrongs can occur independent of and in addition to harm-based-wrongs, and the reaction of the affected subject to these non-harm-based wrongs is not an indicator of their badness. Violations of dignity, for example, qualify as wrongs in the sense of a non-harm-based wrong. They constitute a moral problem even if the affected being doesn’t understand that they are being undignified and even if their welfare is good (Hacker-Wright 2007, 453; Cary and Gruen 2022, 83). Suzanne Cataldi, for example, argues a notion of animal dignity that problematizes them being “viewed and treated as (or reduced to) something less or something other than what they are” (Cataldi 2002, 117). Inhibiting the caring capacities or relationships of caring beings, on this account, can be viewed as a violation of dignity, because it fundamentally reduces and warps them into something they are not.

But it is not just the denial of the value of care in animals that leads to violations of dignity here, but also its (partial) recognition by welfarism. As Alice Crary and Lori Gruen (2022, ch. 5) point out, in the context of capitalism, violations of animal dignity also manifest in the complete utilization, or exploitation, of every part of the animal. Care has been discovered as one of those usable parts, as highly *instrumentally valuable* for human gains. We see animals exploited and instrumentalized *as caregivers* in industries that are antithetical to care, e.g. in the dairy and meat industry, *because* care improves welfare. Thus, this instrumentalization implies at least partial recognition of the value of care. At the same time, the incorporation of “dignity” into animal welfare law terminology often seems reductionist (Kurki 2023) and further obfuscates the limitations of an ethical perspective that only deems wrong what causes experiential harm. Accordingly, there is renewed interest in the notion of animal dignity as independent of subjective experience (Challenger 2023).

By help of Martha Nussbaum’s (2004; 2007) capabilities approach we can also say that a caring individual cannot flourish as the kind of *moral* individual she is if her care is thwarted. Again, this is not just a problem in terms of welfare but of dignity and justice (Nussbaum 2004; 2007). Core capabilities like “to love those who love and care for us” or to “live with concern for and in relation to others” need “opportunities to function” at least to a “threshold level” in order to lead a life “worthy of […] dignity” (Nussbaum 2008). Indeed, the role of care in one’s moral becoming is a related aspect that we view as another source of the objective moral value of care; we turn to it in the next subsection.

#### The importance of care for the development of caring capacities

The objective value of care is also evident in a specific function of care that we want to emphasize in our argument: its importance in ontogenetic development. Care is fundamental for humans and many other social animals *to become* a social being in the first place. If social beings do not receive care, especially in infancy, they will struggle to show empathy and give care themselves. They will thus lack an important competence for social and ultimately moral interactions, on a care ethical account. This correlation was a fundamental insight in human psychology, ironically through research on animals (Harlow 1958; Harlow et al. 1965; Harlow and Suomi 1971). Maternally deprived monkeys were grossly incompetent in social interactions, they did not initiate or reciprocate play and grooming, and exhibited abnormal, often aggressive, sexual and maternal behaviors (Harlow and Suomi 1971, 1534). Recent studies support these early findings in humans and animals (Haller et al. 2014), including non-primate species like rats (e.g. Veenema and Neumann 2009).

A lack of care in infancy has, furthermore, a long-lasting impact on an individual’s capacity to cope with stress and regulate their own emotions, which are necessary capacities to develop other-regarding concern and the ability to act on it appropriately (Monsó and Wrage 2021). Maternal separation has been linked to “anxiety and depressive-like behaviors in both primates and rodents” (Gilles and Polston 2017, 2). Thus, it is not just objectively valuable states of affairs like love, friendship and trust that are enabled through caring relationships. On an even more fundamental level, receiving and giving care are important for the development of caring beings. This is especially relevant in a care ethical framework that views care as the wellspring of morality; however, other moral frameworks, too, may have to acknowledge care as the biological root of moral concern (Waller 1997). We think this role of care in creating caring, and on some accounts potentially moral beings, and thus in enabling the continued existence of a caring world as we value it is also part of the objective value of care.

## Harm-based and non-harm-based wrongs in our treatment of caring animals

In light of the described value of care and the empirical data on caring animals we need to seriously reconsider our treatment of animals. We will have to consider harm-based wrongs that are subjectively experienced as bad. But we will also have to consider non-harm-based wrongs because of the objective value of care. This includes wrongs that can potentially result from a romanticized valorization of care that neglects matters of justice: the often unjust distribution of care, the potentially reduced autonomy of caregivers, and the possible exploitation of individuals or groups who are (deemed) especially caring (Collins 2015, 8f).

There is a wealth of literature from the care ethical tradition devoted to our treatment of animals (e.g. Donovan and Adams 1996, 2007; Gruen 2015). Animals’ own intraspecific relationships of care, however, have only recently come into focus of animal ethics (Monsó et al. 2018; Cooke 2021; Wrage 2022) also, because human interference with caring animals is ubiquitous, especially in systems of use. This concerns laboratory animals, farmed animals and zoo animals, but also companion animals and wildlife. In the following, we map the scope of interferences into the animals’ *exercise of care behavior* (Sec. “Human interference with animals’ exercise of care behavior”) and into *(the development of) their capacity to care* (Sec.” Human interference with animals’ capacity to care”), including short-term and long-term harm-based wrongs. Finally, we turn to possible non-harm-based wrongs caused by such interferences (Sec. “Emphasizing non-harm-based wrongs against caring animals”). While both kinds of wrongs may often occur simultaneously, we want to highlight that this doesn’t mean that they collapse into each other conceptually. Thus, affected animals might be doubly harmed (Monso et al. 2018, 297). Furthermore, there are cases where even interventions *to improve welfare*, or to mitigate harm-based wrongs, *cause* non-harm-based wrongs. We show the instrumentalization of care in farming to be one such intervention where subjective welfare measures and the objective value of care are at odds.

### Human interference with animals’ exercise of care behavior

Humans regularly hinder animals from caring for others. We do so, for instance, by implementing husbandry conditions like single housing, or management procedures like forced (maternal) separation.

*Single-housing* in lab animals is often implemented in the course of presumed standardization, but it has adverse effects on wellbeing, such as anxiety- and depression-like phenomena (e.g. Berry et al. 2012). Even if some single-housed animals do not show clear signs of such effects on their wellbeing, they miss out on all the subjective values of caring relationships. In the case of pets, animal welfare legislation tries to tackle the problem of social isolation by demanding that at least some species with obviously complex social lives are housed socially (in Austria, for example, parrots must be kept at least in pairs).^4^ Furthermore, social isolation is also an issue in zoos. Providing species like elephants, who form strong social bonds, with appropriate group size and group structure is considered paramount for welfare. Stereotypic behavior in elephants increases with their time spent in single-housing (e.g. Greco et al. 2016). Still, some zoo animals are even permanently single-housed, as in the case of elephant Happy at the Bronx Zoo (NhRP 2022).

This is not to say that social housing is entirely unproblematic: Ratuski and Weary (2021) found that rat dams choose to spend time away from their pups when able to do so, particularly later in lactation; not offering them the opportunity to do so resulted in increased passive nursing and negative affect. In industrial farming, this lack of freedom to disengage from others is a main problem due to overcrowding. Aside from the host of welfare issues it creates, overcrowding also hinders autonomy in building and navigating relationships, because it affords little to no control over who to engage or disengage with. This is as much of a problem to caring animals as social isolation. So, to be considerate of caring animals does not mean to provide them with company all the time. It means to give them (at least some degree of) autonomy in their social lives.

*Forced (maternal) separation* also prevents animals from exercising care behavior. It occurs routinely in farming. Most dairy calves are separated from their mothers at birth and reared individually by help of automatic calf-feeders and bucket feeding (Costa et al. 2016, 2454). The separated animals call for each other for days (e.g. Johnsen et al. 2015; Marchant et al. 2002). Besides forced weaning, gestation crates, farrowing crates, and tethered housing all prevent farmed animals from exercising (the full range) of care behavior towards partners or children (Monsó et al. 2018). In addition, farmed animals are often re-grouped, which involves forced separation. As long-term familiarity creates preferred social partners, for example in adult dairy cows (e.g. Gutmann et al. 2015), we can safely assume that frequent re-grouping means a loss of, or the complete prevention of close bonds.

Social separation also occurs in the lives of companion animals. Forced weaning and early separation from the mother (at four to eight weeks of age) are common in puppies sold through pet stores or born in commercial breeding establishments. They are stressful and traumatic in themselves and make infants less resilient, because they lose the stress buffering effects of their mother and siblings (e.g. McMillan 2017, 23). Still, many people prefer to get pets as young as possible, because they are under the misguided impression that this facilitates human-animal bonding (Slabbert and Rasa 1993).

In zoos some animals are also taken from the care of their mothers to hand-rear them by human staff. Nurseries devoted to hand-rearing infant mammals became a prominent feature in zoos in the 1950 and 1960s (Ogden and Kasielke 2001), and some zoos have followed a policy of automatically hand-rearing animals for a long time, especially high-profile primates like great apes (Porton and Niebruegge 2006). Zoos also frequently exchange animals between institutions for the sake of conservation breeding, thereby forcing separation. This is furthermore also part of conservation strategies and ‘population management’ in the wild. Humans heavily interfere with wild animals’ social bonds and communities by kidnapping, relocating and sometimes slaughtering individuals or entire parts of communities. This is exemplified by large-scale ‘culling’ of African elephants from the 1960 to 1990s to compensate for habitat loss (Bradshaw et al. 2005).

Across contexts of human-animal interaction, humans disrupt and prevent animals’ relationships. Our interferences often entail acute harm-based wrongs but they likely also have a more long-lasting, global and possibly permanent effect on the individual or entire groups and populations, which we turn to in the next subsection.

### Human interference with animals’ capacity to care

Forced social separation and maternal deprivation do not only prevent caring animals from exercising care behavior in the moment. They can, in addition, temporarily or even permanently inhibit the animals’ *capacity* to care because receiving care plays a significant role in the development of caring capacities. Again, interferences with animals’ capacity to care can be observed in different contexts of animal use, resulting in a range of subjective harms. They usually happen as a side-effect of specific practices, but have also been inflicted purposefully in animal experimentation.

Studies in neuropsychology subject countless animals, foremost rodents, but also primates, to procedures that *directly and purposefully* diminish their caring capacities, e.g. by inflicting amygdala lesions or hippocampus damage (see e.g. Hernandez-Lallement et al. 2018; Bliss-Moreau et al. 2013; Moadab et al. 2015). To the same effect, maternal deprivation studies, very much in the spirit of Harlow’s (1958) pioneering research, are conducted to this day (e.g. Tóth et al. 2008; Tulogdi et al. 2014). Usually, the objective of this research is to create animal models of human psychopathology, like conduct disorder, characterized by callousness, a lack of empathy and pro-social behavior, abnormal aggression, and other social deficits (Hernandez-Lallement et al. 2018; Macrì et al. 2018). These animal models are then used to study the impact and reversibility of these disorders, and the effects of possible therapies and medication for human patients. The animals created can no longer build caring relationships with conspecifics.

Humans do not only purposefully interfere with animals’ capacity to care in labs. The same damage also occurs as a by-product when *long-term effects* of social isolation or maternal separation are ignored or accepted. Puppies who show increased aggression, fear or other emotional and behavioral problems in adulthood as a result of early weaning are ultimately inhibited in their learning of social norms and thus their very social capacity (McMillan 2017; Pierantoni et al. 2011). The same holds for hand-reared zoo animals, who run the risk of losing defining social touch patterns like grooming, or maternal abilities as adults (Ryan et al. 2002; Porton and Niebruegge 2006; Freeman and Ross 2014; Kalcher-Sommersguter et al. 2015). In farmed animals we also find a range of long-term effects of maternal separation, such as diminished exploratory behavior and learning (Weary et al. 2008; Costa et al. 2016; Mandel and Nicol 2017; Beaver et al. 2019).

Additionally, wild animals who are separated from their social partners or family due to human interference also suffer negative long-term effects on their capacity to care. This is exemplified by the effects of ‘culling’ in elephants. These animals live in complex social communities heavily reliant on experienced elders (Poole and Moss 2008). They also seem to mourn their dead (Goldenberg and Wittemyer 2020). ‘Culling’ disrupts elephant communities long-term by eradicating entire older generations within family units, leaving young elephants, who were routinely spared, without this vital social support. Bradshaw et al. (2005) describe that the severely traumatized male infant survivors of such mass slaughter exhibit heightened intra- and interspecies violence in their adolescence, in one park killing around 100 rhinos, something that was unheard of before. This rampage stopped immediately after older males were reintroduced into the population, likely providing the deviants with the social guidance they were deprived of, as they missed out on the secondary socialization they would have experienced if they had joined a bull herd at the appropriate age.

### Emphasizing non-harm-based wrongs against caring animals

The issue of harm-based wrongs caused by the inhibition of care behavior and the capacity to care in animals is acknowledged by a body of literature in animal welfare science, as we have shown above. The fact, however, that ethical theory also points to an objective value of care is rarely addressed. To counter this, we first show what we miss when reducing our ethical evaluation of interferences with caring animals to a narrow notion of subjective welfare. Second, we give some examples of interventions to mitigate harm-based wrongs in caring animals that actually *cause non-harm-based wrongs*, as they instrumentalize care and facilitate the further instrumentalization of caring animals as such.

One example that shows that we cannot reduce our ethical evaluation of interferences with caring animals to welfare is provided by research that permanently inhibits or destroys animals’ capacity to care, e.g. by social deprivation or amygdala lesioning. This is because this research fundamentally changes what welfare even means for the beings it produces, which may suggest that they are not being harmed. Callousness may be induced painlessly and the animals become indifferent to others and to their lack of relationships. However, this research is only useful in animals that naturally *did* care, because it seeks to find a cure, a way to reverse the damage. It requires that a once caring animal is diminished in their very nature, their life is impoverished. We acknowledge that this is a worse life to live in humans, because this kind of research is done, precisely, to treat callousness in humans.

This research on animals is unlikely to slow down, as it has been somewhat ‘revolutionized’ by the insight that rodents possess sufficiently similar caring capacities to primates. In a study where rats have been purposefully made callous, the scientists write that this offers a “cheap, convenient, and *ethically less controversial* alternative” to nonhuman primates (Hernandez-Lallement et al. 2018, 4, our emphasis). They fail, however, to provide reasons why it would be ethically preferable to make a caring rat instead of a caring macaque callous, all the while trying to cure callousness in humans. Every caring being whose caring capacities are quenched or diminished is wronged by this. They miss out on the subjective value(s) of care that would make their life richer and possibly meaningful, and they lose access to other valuable states of affairs, like the experience of love or trust. We need at least a broad notion of wellbeing including the deprivation of goods as a harm to address what’s going wrong here. But the objective value of care points to further wrongs. Inducing callousness is a significant infringement on the individual’s social and potentially moral life, and a loss to a world that values care morally. Considering that care plays a fundamental role in the lives of caring animals, making them callous means a complete distortion and total subjugation under human interests. Therefore, not least, it speaks of our own moral character if species membership alone is enough for us to determine whether such an intervention would be a horrific violation or convenient.

We can see from this case that a perspective focused on subjective welfare alone falls short in capturing parts of the problem. This can be further illustrated by the case of ‘culling’ for wildlife population control. We have mentioned before how elephant survivors of such interventions later in life develop PTSD-like issues and become dangerous to their environment. If this is addressed purely in welfare terms without regard for the objective value of care and (networks of) caring relationships, one could come up with solutions like more thorough ‘culling,’ done quickly and painlessly. After all, if the young elephants had not been spared and had not witnessed their families’ violent death, they would not have developed into ‘problem elephants.’ To avoid this issue then, entire families would have to be killed, while others could be left entirely intact. Indeed, there have been policy changes to that effect based on this reasoning (e.g. Zenda 2021). These measures do in principle acknowledge the importance of intact relationships, but only for the purpose of preventing the occurrence of ‘problem elephants,’ not as objectively valuable states of affairs. Leaving behind nobody who cares is not more humane, it just obscures the wrongness of killing caring animals behind welfare concerns. This leads us to the issue of welfare measures that *cause* non-harm-based wrongs against caring animals, or the problem that a welfare perspective may not just overlook but actively deny the objective value of care.

Care in animals is not only disrupted on the individual and relational level, but also intentionally evoked and artificially substituted in contexts of animal use, and thus *instrumentalized* in human-animal interactions. Cooke (2021) has recently problematized this from a rights perspective. Care ethics can help to further capture the wrongs that different forms of instrumentalization of care entail, especially when we acknowledge *moral* care in animals (Wrage 2022). We identify two forms of instrumentalization: The artificial substitution of care to induce desired effects of care, and the instrumentalization of animals as caregivers. Due to space restrictions we limit our discussion to farming, but the instrumentalization of animals as caregivers may also happen, for example, in pet-keeping, animal-assisted interventions, to service animals, and to animals in entertainment who do emotional labor.^5^

Artificial substitutes for care in farming range from cattle brushes that are offered to (at least partially) replace social grooming (e.g. Velasquez-Munoz et al. 2019) to the administration of hormones that are associated with attachment to facilitate forced weaning (e.g. Rault et al. 2015). The ‘soothing’ provision of artificial care or ‘care substitutes’ ascribe a mere instrumental value to care in contexts where actual relationships are prevented or disrupted. They aim to produce the *functional outcome* of care to the degree that is useful to these systems, e.g. to facilitate handling, or help continued weight-gain in freshly weaned animals. Providing such substitutes amounts to an instrumentalization of care, because its positive effects on the animals are sought out but entirely detached from actual relationships.

Moreover, the instrumental value of care to systems of animal use is sought via the *instrumentalization of caring animals*. Dairy farming faces the dilemma of needing mothers (to produce milk) and children (to trigger milk production, and to be used as dairy cows later on), but a basic part of the mother–child relationship, nursing, is counterproductive to the objective of dairy farming, as it lowers milk yield. In turn, entirely preventing cow-calf relationships is associated with behavioral and handling issues, and has welfare costs that have long-term productivity costs in the offspring, such as slower weight gain (Meagher et al. 2019). Some dairy farmers thus move from motherless to fostered calf-rearing. In such systems one foster cow usually nurses two to four calves without being milked herself (e.g. Hudson 1977; Johnsen et al. 2016). This secures high levels of milk yield from the birth mothers, while allowing some natural behavior in foster cows and calves. However, while most foster cows accept other calves, they often show less affiliative behaviors towards them compared to their own calves (Johnsen et al. 2016). Moreover, this allegedly more welfare-friendly management of dairy cows still leaves behind the birth mothers suffering the loss of their babies (Flower and Weary 2001), while forcing them to continuously exhibit the one parental behavior they can be exploited for, lactation.

Aside from the obvious harm-based wrongs this practice still entails, it also shows a fundamental disregard for the objective value of care. Cows are, as we have argued, empathic caregivers and form deep social bonds. However, their caring is treated as a commodity that can be harnessed and provided efficiently to the extent that it is instrumentally valuable to the system of use. Their (potential) relationships are not viewed as particular or to be treated with respect as morally valuable states of affairs or important to those involved. Instead, care merely greases the wheels of a fundamentally uncaring industry. In a final step, this more welfare-friendly approach is also suited to improve the image of dairy farming and thus sustain consumer acceptance of dairy products in general, because it can offer ‘wholesome’ images of (foster) cow-calf bonds, instead of the off-putting images of isolated calves in calf igloos the industry has a reasonable interest in hiding. The foster cows who are assigned the caregiver role can thus be instrumentalized in yet another way, namely to obscure the wrongs that are inherent to this form of animal use. Purposefully exploiting a phenomenon that has inherent value and reducing it to its instrumental value, again, should worry us beyond welfare considerations as a non-harm-based wrong. Indeed, such wrong-doing can, by help of care-ethical reasoning, be framed as a profound injustice. Nussbaum (2007, 397–398) argues that animals are “entitled” to lives in which they can have attachments to others, and in which they can engage in interrelationships with others that care and love back (Nussbaum 2007, 397–398, our emphasis). Any thwarting of such relationships or reduction of their quality amounts to an instance of injustice (Nussbaum 2004, 2007).

## Outlook

We have argued that many animals have caring relationships – the kind of empathic relationships we value in humans in our everyday lives and in ethical theory. Still, the value of care in animals, the moral status of caring animals, and possible ethical implications for their treatment have been largely neglected in scholarly debate and in society. Interferences with animals’ caring relationships and the development of their caring capacities occur regularly and systematically, especially in systems of animal use, and are problematized, if at all, in narrow terms of individual welfare. We have argued the non-harm-based wrongs in addition to harm-based wrongs these practices cause to provide us with a better idea of how some animals might be ‘doubly wronged’ (Monsó et al. 2018, 297).

We found that care has subjective and objective value and thwarting it can cause harm-based as well as non-harm-based wrongs. Harm-based wrongs consist of immediate welfare harms or a deprivation of future goods (like trust or friendships), because such goods are instrumentally valuable for wellbeing and their deprivation leads to suffering. All of this can be addressed from a broad welfare perspective. Non-harm-based wrongs, however, are non-experiential in nature and point to values other than welfare, such as the objective value of care, the role of care in the context of flourishing, or the dignity of caring animals. The instrumentalization of care and caring animals provides one example for non-harm-based wrongs that even an expansive welfare concept cannot address.

The recognition of the multi-faceted value of care in animals as we have described it here adds to the criticism of traditional human-animal relations rooted in dominion. The case of care also highlights that even with scientific recognition of ‘sufficiently human-like capacities’, there is a stubborn lag of ethical implementation. In the meantime, preventable wrongs are done. While we have spent most of our time considering human wrongdoing in this paper, we want to conclude by highlighting that a recognition of the value of care in animals is, maybe most importantly, an uncovering of good in the world: Caring animals are co-authors of a more ethical world, and we will all gain by engaging with them as such.

## Footnotes

1 This kind of non-reflective intentionality attributed to animals has, for example, been defended by Waller (1997), Sebo (2017), Wilcox (2019), or Wrage (2022). They suggest that between non-voluntary, automatic behavior (such as a seagull’s reflexive regurgitation of food for their young) and highly reflective intentional action (such as the writing of a paper), there is a vast realm of non-reflective behavior that is purposeful and flexible, and thus has some features of intentional/agential behavior. Both human and nonhuman animals frequently engage in these behaviors. Examples are spontaneous emotionally motivated behaviors (such as consoling a friend), or purposeful, well-practiced behaviors that don’t require reflection *anymore* (skills like burping a baby).

2 Care ethics, while still marginalized, points to something important missed by mainstream moral theory with its focus on the autonomous individual. This is the importance of care as a fundamental *moral practice*, and the normative weight of caring relationships. However, while care may precede the possibility of justice due to its role in moral development, care without justice is also undesirable (Bubeck 1995; Collins 2015, 5). We take it that care, while fundamental to a moral society, needs to be complemented by justice and vice versa. Care is thus one among several important moral capacities.

3 This interpretation of flourishing as a notion going beyond welfare can further be based on the Aristotelian background of Nussbaum’s theory, specifically Aristotle’s idea from the Nicomachean Ethics (1.7) that there is such thing as an *ergon*, a characteristic activity of a being that is tightly linked to what the good is for that being.

4 See Sect. "Empirical data on empathic care in animals" of the Appendix 2 of the so-called 2. Animal Husbandry Act (available online: https://www.ris.bka.gv.at/GeltendeFassung.wxe?Abfrage=Bundesnormen&Gesetzesnummer=20003860, 13/07/2022).

5 By which we mean that these animals are used (often also trained) to show behavior that is supposed to elicit positive emotions in human viewers (Beardsworth & Bryman 2001, 98).
